# Spatiotemporal Pattern of COVID-19–Related Mortality during the First Year of the Pandemic in Brazil: A Population-based Study in a Region of High Social Vulnerability

**DOI:** 10.4269/ajtmh.21-0744

**Published:** 2021-11-10

**Authors:** Lucas Almeida Andrade, Wandklebson Silva da Paz, Alanna G. C. Fontes Lima, Damião da Conceição Araújo, Andrezza M. Duque, Marcus Valerius S. Peixoto, Marco Aurélio O. Góes, Carlos Dornels Freire de Souza, Caíque J. Nunes Ribeiro, Shirley V. M. Almeida Lima, Márcio Bezerra-Santos, Allan Dantas dos Santos

**Affiliations:** ^1^ Nursing Graduate Program, Federal University of Sergipe, Aracaju, Brazil;; ^2^ Collective Health Research Center, Federal University of Sergipe, Aracaju, Brazil;; ^3^ Parasitic Biology Graduate Program, Federal University of Sergipe, Aracaju, Brazil;; ^4^ Tropical Medicine Graduate Program, Federal University of Pernambuco, Recife, Brazil;; ^5^ Health Sciences Graduate Program, Federal University of Sergipe, Aracaju, Brazil;; ^6^ Department of Occupational Therapy, Federal University of Sergipe, Lagarto, Brazil;; ^7^ Department of Speech Therapy, Federal University of Sergipe, Aracaju, Brazil;; ^8^ Department of Medicine, Federal University of Sergipe, Aracaju, SE, Brazil;; ^9^ Sergipe State Department of Health, Aracaju, Brazil;; ^10^ Department of Medicine, Federal University of Alagoas, Arapiraca, Brazil;; ^11^ Departament of Nursing, Federal University of Sergipe, Lagarto, Brazil;; ^12^ Departament of Morphology, Federal University of Sergipe, Aracaju, Brazil

## Abstract

Currently, the world is facing a severe pandemic caused by the new severe acute respiratory syndrome coronavirus 2 (SARS-CoV-2) virus. Although the WHO has recommended preventive measures to limit its spread, Brazil has neglected most of these recommendations, and consequently, our country has the second largest number of deaths from COVID-19 worldwide. In addition, recent studies have shown the relationship between socioeconomic inequalities and the risk of severe COVID-19 infection. Herein, we aimed to assess the spatiotemporal distribution of mortality and lethality rates of COVID-19 in a region of high social vulnerability in Brazil (Northeast region) during the first year of the pandemic. A segmented log-linear regression model was applied to assess temporal trends of mortality and case fatality rate (CFR) and according to the social vulnerability index (SVI). The Local Empirical Bayesian Estimator and Global Moran Index were used for spatial analysis. We conducted a retrospective space–time scan to map clusters at high risk of death from COVID-19. A total of 66,358 COVID-19–related deaths were reported during this period. The mortality rate was 116.2/100,000 inhabitants, and the CFR was 2.3%. Nevertheless, CFR was > 7.5% in 27 municipalities (1.5%). We observed an increasing trend of deaths in this region (AMCP = 18.2; *P* = 0.001). Also, increasing trends were observed in municipalities with high (*N* = 859) and very high SVI (*N* = 587). We identified two significant spatiotemporal clusters of deaths by COVID-19 in this Brazilian region (*P* = 0.001), and most high-risk municipalities were on the coastal strip of the region. Taken together, our analyses demonstrate that the pandemic has been responsible for several deaths in Northeast Brazil, with clusters at high risk of mortality mainly in municipalities on the coastline and those with high SVI.

## INTRODUCTION

In the past year, the world has been facing a new viral infection caused by the severe acute respiratory syndrome coronavirus 2 (SARS-CoV-2). The first cases emerged in the city of Wuhan (Hubei Province, China), and since then, the virus has spread quickly on a large scale in several countries. The disease, termed COVID-19, was declared a pandemic by the WHO in March 2020 and has been causing significant social, economic, political, and public health impacts worldwide.^1^

Despite all the efforts and strategies instituted in several countries to contain the pandemic, the number of cases and COVID-19–related deaths is still increasing. Currently, more than 200 countries are affected by the pandemic.^2^ In addition, failures and delays in the immediate implementation of measures to combat the pandemic occurred in some countries, resulting in catastrophic impacts on public health, especially in the United States and Brazil.^3^

According to the WHO, up to May 2021, 157.7 million cases and approximately 3.2 million deaths from COVID-19 were recorded worldwide.^2^ Since the first confirmed case in Brazil on February 26, 2020,^4^ the country has accumulated 21 million cases and more than 590,000 deaths, ranking second in the world in the number of deaths (behind the United States).^2^ Importantly, Northeast Brazil has the second largest number of cases in the country, with a total of 4.7 million cases and 116,000 deaths.^5^ Conversely, this region has one of the worst socioeconomic indicators in the country.^6^

Considering the spatial distribution of cases, the use of geographic information systems (GIS) and spatial analysis techniques allows the mapping and identification of high-risk areas and assists health services in the planning and implementation of health measures to control the disease.^6^ In this context, several studies using spatiotemporal analysis tools have demonstrated the impact of morbidity, mortality, and geographic spread of COVID-19 worldwide.^7^^–^^10^ Importantly, a study carried out in the United States identified COVID-19 spatiotemporal clusters, which were classified as priority areas for resource allocation and implementation of disease-control measures.^11^

Likewise, studies assessing the spatiotemporal patterns of COVID-19 have been developed extensively in Brazil.^6^ A prior study conducted by our group evaluated the spatiotemporal patterns of SARS-CoV-2 in northeast Brazil, where high-risk clusters were identified, mainly in the states of Ceará and Maranhão. Additionally, we demonstrated the dispersion of cases from metropolitan areas to inland municipalities.^12^

The dispersion of COVID-19 has a heterogeneous dynamic across Brazilian regions. Regardless of the SARS-CoV-2 infection having the potential to reach all communities, some areas are noticeably more affected by virtue of the socioeconomic determinants and health services available.^13^ Furthermore, measures to control the virus are more difficult in areas of high social vulnerability, such as slums and inland municipalities.^13^^,^^14^

Notably, Northeast Brazil has precarious socioeconomic indicators, such as a low municipal human development index (MHDI) and high social vulnerability index (SVI), with marked social disparity.^15^^,^^16^ Considering the high transmissibility of SARS-CoV-2 and the emergence of the pandemic in Brazil, studies to analyze the disease in space and time are required.^11^ Importantly, the application of spatial modeling methods is useful in monitoring outbreaks and identifying active and emerging clusters during the pandemic.^17^ Herein, we aimed to assess the spatiotemporal patterns of mortality and CFRs from COVID-19 in a region of high social vulnerability (Northeast Brazil) during the first year of the pandemic.

## MATERIALS AND METHODS

### Study design, population, and period.

We conducted an ecological and population-based study, using spatial and temporal techniques to assess the mortality and CFR due to COVID-19 in the municipalities of Northeast Brazil. The social vulnerability indicators of the municipalities were associated with data on mortality from COVID-19. The units of analysis were the populations from the 1,794 municipalities in this region. Herein, we analyze all deaths related to COVID-19 confirmed during the first year of the pandemic, from March 27, 2020 to March 27, 2021.

### Study area.

**Figure 1.**
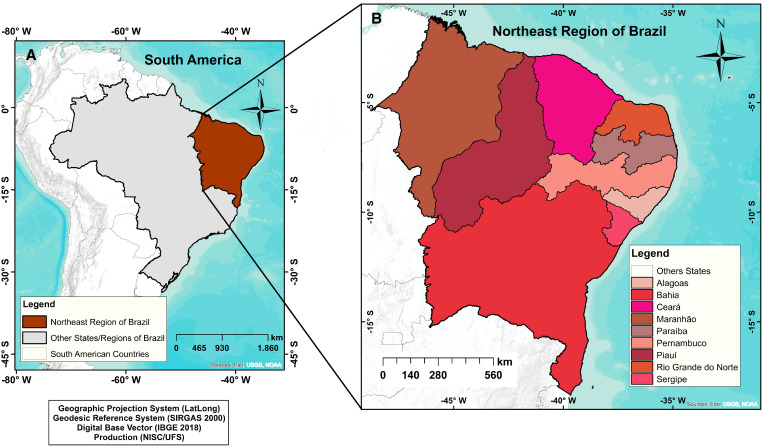
Study area: the states of the Northeast Brazil. This figure appears in color at www.ajtmh.org.

Brazil is divided geographically into five regions: Midwest, South, Southeast, North, and Northeast.^15^^,^^16^ The Northeast region (latitude: 01°02′30″ N/18°20′07″ S; longitude: 34°47′30″ E/48°45′24″ W) has the largest number of federative units, with nine states: Alagoas (102 municipalities), Bahia (417), Ceará (184), Maranhão (217), Paraíba (223), Pernambuco (185), Piauí (224), Rio Grande do Norte (167), and Sergipe (75) (Figure 1); has the third-largest territorial area of the country (1,558,000 km^2^); and a population of 57,071,654 inhabitants (the second most populous region in Brazil), which corresponds to approximately 30% of the Brazilian population. However, the highest demographic density occurs in the cities of the coastal strip of the region.^15^ Importantly, the Northeast region has the lowest Human Development Index in Brazil (0.663),^18^ municipalities with high SVI, and prevalence of several neglected diseases, such as schistosomiasis,^15^ leishmaniasis,^16^ and leprosy.^19^

### Study variables and sources of data.

The epidemiological variables used in this study were as follows: 1) the absolute number of daily and monthly deaths from COVID-19 in the 1,794 municipalities of Northeast Brazil; 2) the mortality rate due to COVID-19. To calculate the mortality rate, we used the number of deaths from COVID-19 as the numerator and the corresponding population as the denominator. It was determined per 100,000 inhabitants; and 3) the CFR due to COVID-19. To calculate the lethality rate, we considered the total number of COVID-19–related deaths as a numerator and the total number of confirmed cases as the denominator. The result was multiplied by the constant of 100 and expressed as percentage (%).

The SVI was also used. This estimates the degree of social vulnerability to which a population is exposed, being composed of three dimensions: urban infrastructure, human capital, and income and work. The SVI ranges from 0 to 1 and is classified as very low (0 to 0.200), low (0.201 to 0.300), medium (0.301 to 0.400), high (0.401 to 0.500), and very high (≥ 0.501).^20^

Data on COVID-19–related deaths were extracted from the surveillance database of the Brazilian Ministry of Health (https://covid.saude.gov.br/). Population estimates for states and municipalities were collected from the Brazilian Institute of Geography and Statistics (IBGE) (https://www.ibge.gov.br/), considering the intercensus estimates for 2020. Social indicators (SVI) were collected from the Brazilian Social Vulnerability Atlas (http://ivs.ipea.gov.br/index.php/pt/). Finally, for spatial analysis, the digital cartographic grid of the Northeast region, divided by municipalities and in shapefile format, was obtained from the Geographic Projection System latitude/longitude (Geodetic Reference System, SIRGAS 2000).

### Time trends analysis.

Initially, we performed a descriptive analysis of the absolute number of deaths and the mortality rate for the Northeast region and states during the first 12 months of the pandemic. Next, to analyze the time trend of COVID-19–related deaths, we carried out a segmented log-linear regression model, using joinpoint regression models. The time trend of deaths from COVID-19 in the municipalities, classified according to the SVI and their respective subdomains, was also analyzed. The Monte Carlo permutation test was applied to select the best model for inflection points (with 999 permutations) and considering the highest residue determination coefficient (*R*^2^). In addition, to describe the time trends, we calculated the monthly percentage changes (MPCs) and their respective 95% confidence intervals (CIs).^21^ If more than one significant inflection was detected during the study period, the average monthly percentage changes (AMPCs) were also calculated. Time trends were considered statistically significant when the MPC or AMPC had a *P* value of < 0.05 and their CI 95% did not include zero. Here, a positive and significant MPC value indicates an increasing trend; conversely, a negative and significant MPC indicates a decreasing trend, and nonsignificant trends are described as stable, regardless of MPC or AMPC values.^22^

### Spatial cluster analysis.

First, we performed the spatial distribution of COVID-19 mortality rate and CFR in the general population. After that, the Local Empirical Bayesian Estimator was used to smooth the crude mortality rate by correcting for instability caused by the random fluctuation of deaths in space.^23^ All rates were represented on choropleth maps, which were stratified into five categories of equal intervals.

Subsequently, to verify whether the spatial distribution of mortality and CFR by COVID-19 occurred randomly in space, the spatial autocorrelation analysis was used by calculating the Univariate Moran Global Index, which ranges from –1 to +1. Values between 0 and +1 indicate positive spatial autocorrelation, values between –1 and 0 indicate negative spatial autocorrelation, and values that cross zero indicate spatial randomness.^23^^,^^24^

Finally, the Moran Local Index (Local Spatial Association Index [LISA]) was calculated to identify areas with spatial dependence and their relationship with neighbors. From there, a scatter diagram was established with the following spatial quadrants: Q1 (high/high) and Q2 (low/low), which indicate municipalities with similar values to those of their neighbors and with positive spatial association; Q3 (high/low) and Q4 (low/high) indicate municipalities with different values from those of their neighbors, with no spatial association. Significant results were represented on Moran maps,^23^^,^^24^ and data were considered statistically significant if a *P* value of < 0.05 was obtained.

### Retrospective spatiotemporal cluster analysis.

A retrospective space–time scan analysis was performed to identify high-risk clusters for COVID-19-related deaths, using the Poisson probability distribution model.^17^^,^^25^ This method allows the mapping of clusters that occur in the space and time.^6^ Our null hypothesis (H0) was that the expected number of deaths by COVID-19 in each area is proportional to the size of its population, while the alternative hypothesis (H1) was that the number of deaths exceeds the expected number of deaths derived from the null model.

Then, we ran the cluster analysis model, considering the following parameters: minimum aggregation time of 1 month, minimum of five deaths, no overlapping of clusters, circular clusters, maximum size of the spatial cluster of 10% of the population at risk, and maximum size of the temporal cluster of 50% of the study period.^6^ The main and secondary clusters were detected using the log-likelihood ratio (LLR) test and represented in thematic maps. We also calculated the relative risk (RR) of death from COVID-19, considering each municipality and agglomerates in relation to their neighbors. Results were considered statically significant when *P* values of < 0.05 were obtained using 999 Monte Carlo simulations.^26^

### Software.

Microsoft Office Excel 2010 software (Microsoft Corporation; Redmond, WA) was used for data tabulation and descriptive analysis; Joinpoint Regression Program version 4.2.0 (Statistical Methodology and Applications Branch, Surveillance Research Program National Cancer Institute, Bethesda, MD) was applied for time-trend analysis^27^; QGis version 3.4.11 (QGIS Development Team; Open-Source Geospatial Foundation Project, CC BY-SA, Las Palmas, CA), was used to generating the choropletic maps^28^; TerraView version 4.2.2 (Instituto Nacional de Pesquisas Espaciais, INPE, São José dos Campos, Brazil) was used for spatial analysis^29^; and SaTScan version 9.6 (Harvard Medical School, Boston, MA and Information Management Service Inc., Silver Spring, MD) was applied for spatiotemporal scanning and cluster analysis.^26^

### Ethical considerations.

Herein, we used public-domain aggregate secondary data and followed national and international ethical recommendations, as well as the rules of the Helsinki Convention. There was no way to identify the subjects; therefore, the use of informed consent was dispensed with. The project was approved by an ethics and research committee: CAAE n. 14384719.8.0000.5546.

## RESULTS

**Figure 2.**
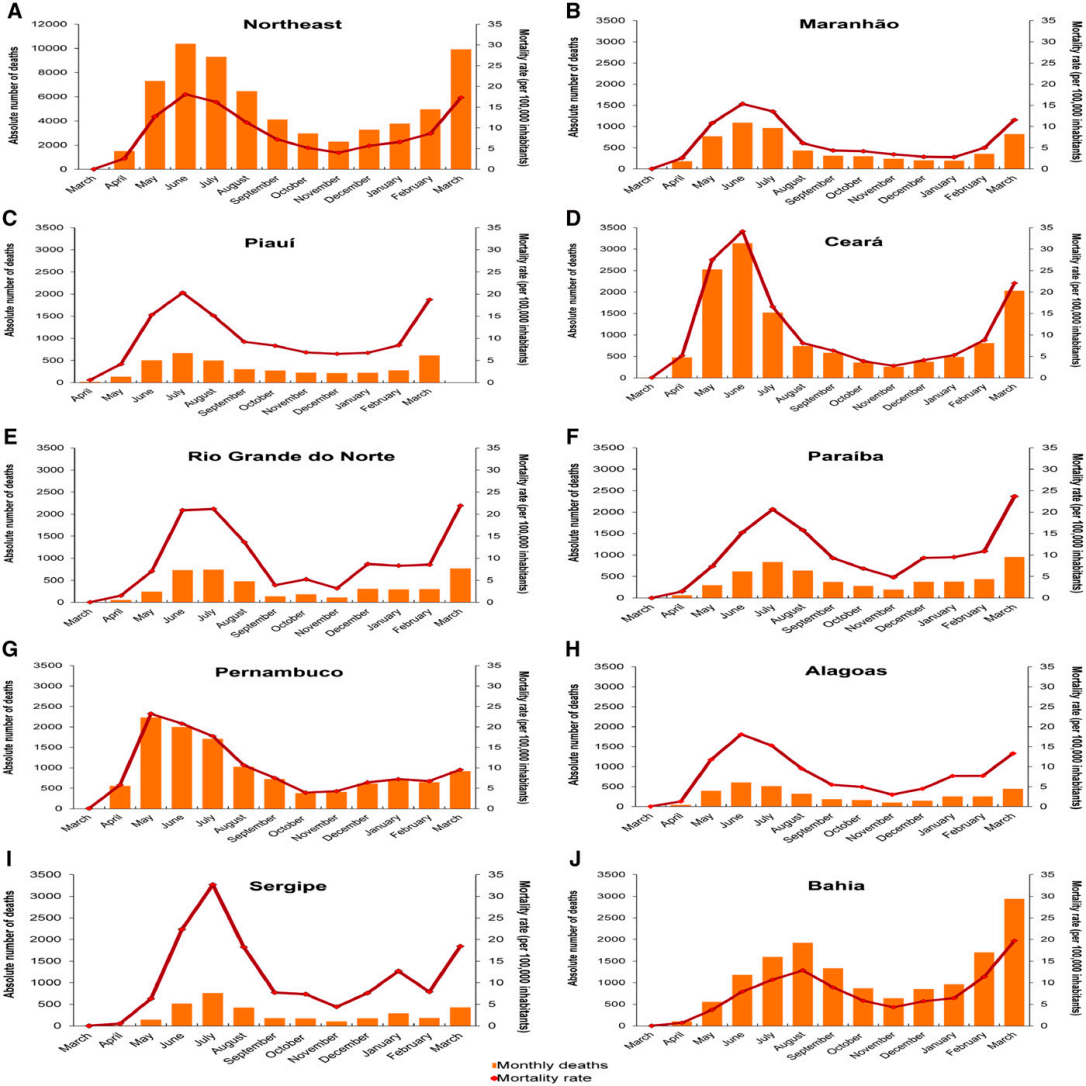
Monthly distribution of the absolute number of deaths and mortality rate due to COVID-19 in the states of Northeast Brazil, 2020–2021. This figure appears in color at www.ajtmh.org.

A total of 66,358 COVID-19-related deaths occurred in Northeast Brazil during the first year of the pandemic. The mortality rate was 116.2 per 100,000 inhabitants, while the CFR was 2.3%. The states with the highest number of deaths were Bahia (*N* = 14,667), Pernambuco (13,733), and Ceará (13,313). We observed that the peak of deaths occurred between May and August 2020 (the first wave of the pandemic) in all states, with a progressive reduction in the following months (Figure 2). Nonetheless, some states also showed a marked increase in mortality rate in March 2021, considered the second wave of the pandemic in Brazil. Considering the states, the highest mortality rates were observed in Sergipe and Ceará (147.4 and 145.7/100,000, respectively). The state of Alagoas showed the lowest mortality rate in the region (103.1/100,000).

In addition to assessing the number of deaths, we also analyzed the time trends of COVID-19-related deaths in Northeast Brazil (Table 1). Interestingly, the segmented linear regression model showed three distinct temporal patterns for this region: first, there was an increasing trend in mortality rate between March and June 2020 (MPC = 117.2; *P* value = 0.04); there was a decreasing trend between June and November 2020 (MPC = –29.6; *P* value = 0.001); and lastly, there was an increasing trend between November 2020 and March 2021 (MPC = 43.3; *P* value = 0.04). Otherwise, when considering the total period (AMPC), seven states showed stable trends in mortality rate. Nevertheless, the states of Bahia (AMPC = 29.7; *P* value = 0.001) and Piauí (AMPC = 49.1; *P* value = 0.001), as well as the Northeast region (AMPC = 18.2; *P* value = 0.001), showed an increasing trend in this period.

**Table 1:** Time trend of mortality rates due to COVID-19 in the states of Northeast Brazil, 2020–2021

Federative unit	Segmented period	MPC (CI 95%)	*P* value	Trend	AMPC (95% CI)	*P* value	Trend
Northeast (region)	3/2020–6/2020	117.2 (2.5 to 360.4)	0.045	↑	18.2 (0.9 to 38.6)	< 0.001	↑
	6/2020–11/2020	–29.6 (–40.7 to –16.4)	0.003	↓			
	11/2020–3/2021	43.3 (21.3 to 69.3)	0.003	↑			
Maranhão	3/2020–6/2020	99.4 (–24.9 to 429.5)	0.129	Stable	14.5 (–7.7 to 42)	0.218	Stable
	6/2020–12/2020	–29.9 (–41.5 to –16.1)	0.004	↓			
	12/2020–3/2021	75.4 (12.6 to 173.1)	0.022	↑			
Piauí	3/2020–6/2020	405.5 (73.8 to 1370.3)	0.011	↑	49.1 (20.4 to 84.5)	< 0.001	↑
	6/2020–1/2021	–16.7 (–22.8 to –10.1)	0.002	↓			
	1/2021–3/2021	83.0 (18.9 to 181.6)	0.015	↑			
Ceará	3/2020–5/2020	695.3 (–97.2 to 224,703.9)	0.388	Stable	33.5 (–35.2 to 174.8)	0.433	Stable
	5/2020–11/2020	–36.3 (–45.3 to –25.8)	0.001	↓			
	11/2020–3/2021	66.0 (30.8 to 110.8)	0.003	↑			
Rio Grande do Norte	3/2020–6/2020	281.6 (–57.4 to 3315)	0.177	Stable	37.1 (–11.9 to 113.4)	0.162	Stable
	6/2020–11/2020	–30.6 (–52.6 to 1.5)	0.056	Stable			
	11/2020–3/2021	49.3 (8.7 to 105)	0.023	↑			
Paraíba	3/2020–7/2020	81.5 (–2.2 to 237)	0.056	Stable	21.5 (–0.3 to 48)	0.053	Stable
	7/2020–11/2020	–31.9 (–55 to 3)	0.063	Stable			
	11/2020–3/2021	45.0 (16.2 to 81)	0.008	↑			
Pernambuco	3/2020–5/2020	377.6 (–96.4 to 63,258.7)	0.448	Stable	19.1 (–36.2 to 122.4)	0.583	Stable
	5/2020–11/2020	–25.4 (–33.5 to –16.2)	0.001	↓			
	11/2020–3/2021	20.0 (0.4 to 43.4)	0.046	↑			
Alagoas	3/2020–6/2020	129.1 (–6.8 to 462.9)	0.064	Stable	18.4 (–1.4 to 42.3)	0.071	Stable
	6/2020–11/2020	–31.1 (–41.3 to –19.2)	0.002	↓			
	11/2020–3/2021	42.2 (20.7 to 67.6)	0.003	↑			
Sergipe	3/2020–7/2020	110.3 (–11.8 to 401.2)	0.079	Stable	20.1 (–10.6 to 61.4)	0.223	Stable
	7/2020–10/2020	–47.7 (–79.8 to 35.5)	0.140	Stable			
	10/2020–3/2021	26.5 (0.3 to 59.6)	0.048	↑			
Bahia	3/2020–7/2020	99.6 (36 to 193)	0.006	↑	29.7 (16 to 45.1)	< 0.001	↑
	7/2020–12/2020	–19.9 (–30.1 to –8.3)	0.008	↓			
	12/2020–3/2021	63.1 (36.8 to 94.4)	0.001	↑			

↑ = increasing; ↓ = decreasing; AMPC = average monthly percentage changes; MPC = monthly percentage changes.

When assessing the time trend of COVID-19–related deaths and considering the SVI of the municipalities, we observed an increasing trend in mortality rates in the municipalities classified as high (AMPC = 19.2; *P* = 0.03; *N* = 859 municipalities) and very high social vulnerability (AMPC = 23.1; *P* = 0.03; *N* = 587; Table 2). Likewise, Domains 2 (income and work) and 3 (human capital) showed an increasing trend in those municipalities classified as having high or very high social vulnerability. Notwithstanding, Domain 1 (infrastructure) showed an increasing trend in municipalities with high, but stability in those with very high social vulnerability.

**Table 2:** Time trend of the mortality rate due to COVID-19 according to the social vulnerability index in municipalities of Northeast Brazil, 2020–2021

	Segmented period	MPC (CI 95%)	*P* value	Trend	AMPC	*P* value	Trend
Social vulnerability index							
Very low	3/2020–10/2020	–18.7 (–18.7 to –18.7)	< 0.001	↓	71.7 (71.7 to 71.7)	< 0.001	↑
(1 city)	10/2020–1/2021	1310.1 (1310.1 to 1310.1)	< 0.001	↑			
	1/2021–3/2021	0.2 (0.2 to 0.2)	< 0.001	↑			
Low	3/2020–6/2020	288.8 (–35.3 to 2234.6)	0.109	Stable	36.7 (–4.5 to 95.6)	0.088	Stable
(32 cities)	6/2020–11/2020	–30.7 (–47 to –9.4)	0.017	↓			
	11/2020–3/2021	45.8 (11.4 to 90.7)	0.015	↑			
Moderate	3/2020–5/2020	473.2 (–81.9 to 18,081.3)	0.251	Stable	31.9 (–15.2 to 105.2)	0.219	Stable
(314 cities)	5/2020–12/2020	–21.7 (–27.1 to –15.9)	< 0.001	↓			
	12/2020–3/2021	67.5 (39.9 to 100.4)	0.001	↑			
High	3/2020–6/2020	126.3 (1.3 to 405.5)	0.047	↑	19.2 (1.1 to 40.6)	0.037	↑
(859 cities)	6/2020–11/2020	–25.0 (–35.4 to –12.8)	0.004	↓			
	11/2020–3/2021	31.4 (13.1 to 52.6)	0.005	↑			
Very high	3/2020–6/2020	165.8 (0.8 to 600.7)	0.049	↑	23.1 (1.3 to 49.6)	0.037	↑
(587 cities)	6/2020–11/2020	–26.3 (–36.5 to –14.5)	0.003	↓			
	11/2020–3/2021	31.3 (12.3 to 53.5)	0.007	↑			
Infrastructure							
Very low	3/2020–6/2020	258.6 (72.8 to 644)	0.006	↑	36.3 (18 to 57.3)	< 0.001	↑
(436 cities)	6/2020–12/2020	–17.3 (–22.4 to –11.7)	0.001	↓			
	12/2020–3/2021	40.4 (23 to 60.3)	0.001	↑			
Low	3/2020–6/2020	224.3 (7.2 to 881.6)	0.041	↑	33.8 (7.2 to 67)	0.01	↑
(503 cities)	6/2020–12/2020	–21.8 (–31.1 to –11.3)	0.004	↓			
	12/2020–3/2021	61.7 (26.9 to 106.1)	0.004	↑			
Moderate	3/2020–5/2020	452.9 (–71.5 to 10,633.7)	0.198	Stable	27 (–13.1 to 85.7)	0.217	Stable
(425 cities)	5/2020–11/2020	–29.7 (–35.7 to –23.2)	< 0.001	↓			
	11/2020–3/2021	47.9 (30.7 to 67.2)	< 0.001	↑			
High	3/2020–6/2020	109.4 (10.5 to 296.9)	0.031	↑	16.0 (0.8 to 33.5)	0.039	↑
(221 cities)	6/2020–11/2020	–35.6 (–45.8 to –23.3)	0.001	↓			
	11/2020–3/2021	55.3 (30.7 to 84.5)	0.001	↑			
Very high	3/2020–6/2020	105.3 (–37.8 to 577.7)	0.182	Stable	13.3 (–12.2 to 46.1)	0.336	Stable
(208 cities)	6/2020–11/2020	–31.6 (–47.9 to –10.2)	0.016	↓			
	11/2020–3/2021	36.2 (2 to 82)	0.041	↑			
Income and work							
Very low	3/2020–10/2020	–18.7 (–18.7 to –18.7)	< 0.001	↓	71.7 (71.7 to 71.7)	< 0.001	↑
(1 city)	10/2020–1/2021	1310.1 (1310.1 to 1310.1)	< 0.001	↑			
	1/2021–3/2021	0.2 (0.2 to 0.2)	< 0.001	↑			
Low	3/2020–6/2020	108.1 (–0.3 to 334)	0.051	Stable	17.6 (–0.2 to 38.7)	0.053	Stable
(8 cities)	6/2020–11/2020	–35.9 (–48.4 to –20.4)	0.003	↓			
	11/2020–3/2021	63.9 (33.1 to 101.7)	0.002	↑			
Moderate	3/2020–5/2020	521.1 (–94.9 to 75,085.7)	0.373	Stable	30.3 (–29.4 to 140.5)	0.398	Stable
(71 cities)	5/2020–11/2020	–28.6 (–37.1 to –18.9)	0.001	↓			
	11/2020–3/2021	47.1 (22.6 to 76.5)	0.003	↑			
High	3/2020–6/2020	175.6 (16.2 to 553.6)	0.029	↑	26.3 (6.1 to 50.5)	0.009	↑
(393 cities)	6/2020–11/2020	–26.1 (–36.2 to –14.3)	0.003	↓			
	11/2020–3/2021	37.6 (19.1 to 59)	0.002	↑			
Very high	3/2020–6/2020	181.4 (–3.5 to 720.7)	0.056	Stable	26.1 (2 to 56)	0.032	↑
(1,320 cities)	6/2020–11/2020	–22.9 (–33.4 to –10.8)	0.006	↓			
	11/2020–3/2021	27.9 (10.3 to 48.3)	0.008	↑			
Human capital							
Very low	3/2020–10/2020	–18.7 (–18.7 to –18.7)	< 0.001	↓	71.7 (71.7 to 71.7)	< 0.001	↑
(1 city)	10/2020–1/2021	1310.1 (1310.1 to 1310.1)	< 0.001	↑			
	1/2021–3/2021	0.2 (0.2 to 0.2)	< 0.001	↑			
Low	3/2020–5/2020	529.8 (–99.6 to 914,959.2)	0.545	Stable	32.7 (–47.7 to 236.7)	0.552	Stable
(4 cities)	5/2020–11/2020	–33.2 (–46 to –17.3)	0.005	↓			
	11/2020–3/2021	70.3 (30.4 to 122.6)	0.004	↑			
Moderate	3/2020–6/2020	100.1 (3.5 to 286.8)	0.043	↑	14.4 (–1.2 to 32.4)	0.072	Stable
(68 cities)	6/2020–11/2020	–33.0 (–44.3 to –19.4)	0.003	↓			
	11/2020–3/2021	46.7 (22.3 to 75.9)	0.003	↑			
High	3/2020–6/2020	169.9 (71.6 to 324.5)	0.002	↑	26.0 (15 to 38.1)	< 0.001	↑
(443 cities)	6/2020–12/2020	–22.9 (–27 to –18.6)	< 0.001	↓			
	12/2020–3/2021	57.3 (40.6 to 76.1)	< 0.001	↑			
Very high	3/2020–6/2020	171.0 (5.8 to 594.3)	0.042	↑	26.0 (4.4 to 52)	0.016	↑
(1,227 cities)	6/2020–11/2020	–21.0 (–31.3 to –9.3)	0.007	↓			
	11/2020–3/2021	27.1 (10.7 to 46)	0.007	↑			

↑ = increasing; ↓ = decreasing; AMPC = average monthly percentage changes; MPC = monthly percentage changes.

**Figure 3.**
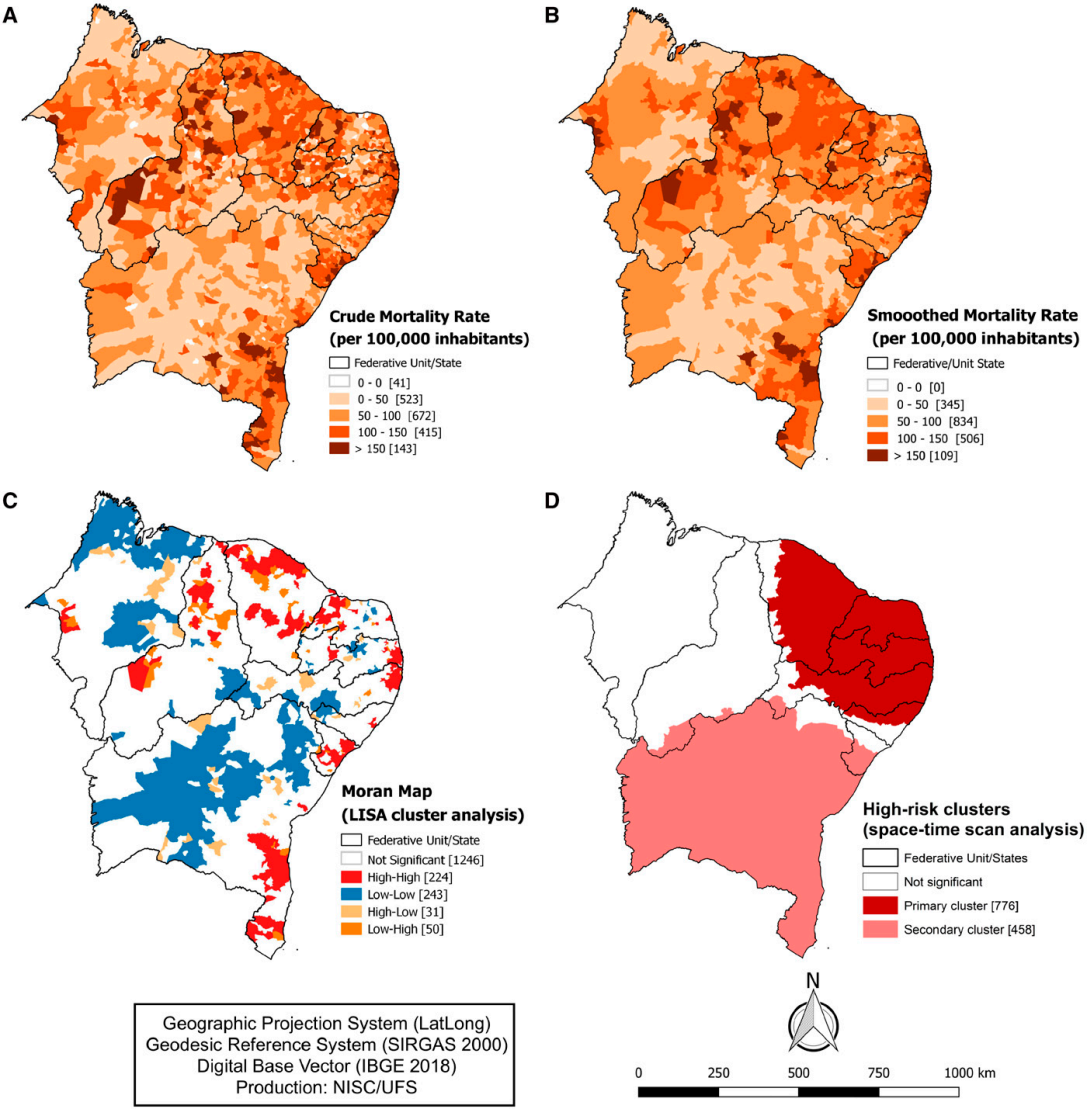
Spatial and spatiotemporal analysis of mortality due to COVID-19 in municipalities of Northeast Brazil, 2000–2021: (**A**) Spatial distribution map according to the crude mortality rates for COVID-19. (**B**) Spatial analysis map according to the smoothed mortality rates. (**C**) Map of spatial autocorrelation analysis by the Local Indicators of Spatial Association (LISA map). (**D**) Map of statistical analysis of spatiotemporal scanning and risk clusters of deaths from COVID-19. IBGE = Brazilian Institute of Geography and Statistics; NISC/UFS = Núcleo de Investigação em Saúde Coletiva, Universidade Federal de Sergipe; SIRGAS = Geocentric Reference System for South America. This figure appears in color at www.ajtmh.org.

The spatial analysis maps showed that deaths from COVID-19 were widely distributed in the states of the Northeast region (Figure 3A and B). About a third of the municipalities (*N* = 558) had crude mortality rates of > 100 per 100,000 inhabitants. These rates were smoothed by the Local Empirical Bayesian Method, and the Moran global index showed significant spatial autocorrelation (I = 0.361; *P* value < 0.001). Furthermore, we identified by LISA analysis the clusters of high risk of mortality formed by 224 municipalities, mostly on the coastline of the Northeast region: Bahia (*N* = 48), Sergipe (33), Piauí (19), Ceará (46), Paraíba (26), Pernambuco (25), Alagoas (5), Maranhão (6), and Rio Grande do Norte (16; Figure 3C).

Additionally, we identified two significant spatiotemporal clusters of COVID-19–related deaths in this Brazilian region (*P* < 0.001; Figure 3D). The primary cluster (between May and July 2020) was formed by 776 municipalities, in the states of Ceará, Rio Grande do Norte, Paraíba, Pernambuco, and Alagoas, with a total of 17,238 deaths and an RR of 2.83 (LLR = 5736.77; *P* < 0.001). On the other hand, the secondary cluster (March 2021) encompassed 458 municipalities in the states of Piauí, Pernambuco, Sergipe, and Bahia, with 3,340 deaths and an RR of 2.30 (LLR = 867.29; *P* < 0.001).

**Figure 4.**
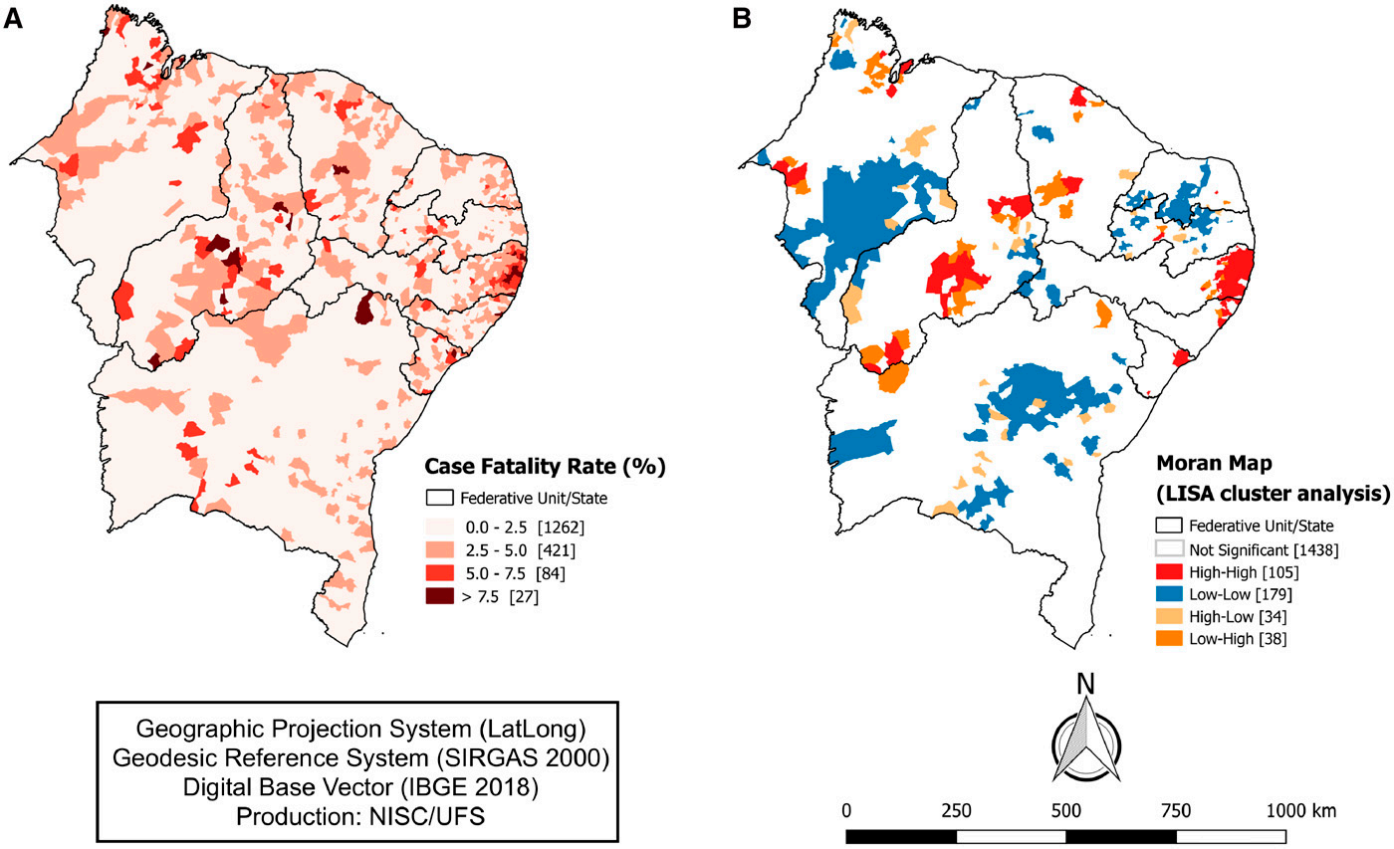
Spatial distribution of municipalities in Northeast Brazil according to the case fatality rate (CFR) by COVID-19, 2000–2021. (**A**) Spatial distribution map according to the gross CFR for COVID-19. (**B**) Map of spatial autocorrelation analysis by the Local Indicators of Spatial Association (LISA map). IBGE = Brazilian Institute of Geography and Statistics; NISC/UFS = Núcleo de Investigação em Saúde Coletiva, Universidade Federal de Sergipe; SIRGAS = Geocentric Reference System for South America. This figure appears in color at www.ajtmh.org.

The spatial distribution of municipalities in the region according to the CFR due to COVID-19 is shown in Figure 4A. As expected, most municipalities had a CFR of between 0 and 2.5% (*N* = 1.262; 70.34%) or between 2.5 and 5% (*N* = 421; 23.46%). Notwithstanding, 84 municipalities (4.68%) had a CFR between 5 and 7.5%, and this rate was > 7.5% in 27 municipalities (1.5%). Moreover, concerning the CFR, the Moran global index showed significant spatial autocorrelation among municipalities (I = 0.264; *P* < 0.001). Correspondingly, the LISA Map showed that the municipalities with the highest CFR (*N* = 105) were located mainly in the states of Piauí, Ceará, Pernambuco, Alagoas, and Sergipe (Figure 4B).

## DISCUSSION

This study provides an overview of the spatiotemporal pattern of mortality and CFR by COVID-19 in a region of high social vulnerability in Brazil during the first year of the pandemic. Notably, our analyses demonstrate that COVID-19-related deaths were distributed widely throughout Northeast Brazil. However, most deaths were concentrated in municipalities on the coast, and the highest mortality rates were observed in the states of Ceará and Sergipe. Furthermore, time-trend analyses showed that the region and the states of Bahia and Piauí exhibited an increasing trend in this period. Importantly, many municipalities had a high CFR, ranging between ≥ 5% and ≥ 7.5%. Taken together, these findings reveal a serious and insidious scenario of the COVID-19 pandemic in Northeast Brazil and highlight that municipalities with high social vulnerability were the most severely affected.

To date, Brazil ranks third in the world in number of cases and second in number of deaths caused by the new coronavirus.^1^ Remarkably, several factors may have contributed to the worsening of the COVID-19 pandemic here. Among them, we highlight social disparities, encouragement in the use of medicines without scientific evidence, delays in the acquisition of vaccines, the unstable political scenario, the mismatch between governments in the implementation of social distancing measures, and the difficulty of managing the health system.^30^

Temporal trend analyses showed an increase in the number of deaths from COVID-19 in Northeast Brazil. Importantly, this region is the second most populous in Brazil and contains important tourism areas. Additionally, it has municipalities with low HDI and high SVI.^15^ Altogether, these characteristics configure this region as a potential risk area for the spread of and mortality by COVID-19. The alarming results observed herein reveal a serious and concerning scenario of the pandemic and demonstrate the urgency of implementing control measures in the most affected municipalities.

Furthermore, the temporal trend analyses showed increasing and decreasing trends in deaths over the first year of the pandemic. Importantly, the Northeast region had been facing difficulties since the beginning of the pandemic, due mainly the high occurrence of severe cases and the reduced capacity of the health services. Moreover, Brazil has been affected by a serious scenario of instability and political polarization.^31^^,^^32^ Concerning this, conflicting measures by state governors and the federal government may have compromised the population’s adherence to the measures of social distancing and prevention measures against the virus, resulting in an even more unstable situation.

On the other hand, most trades were affected by the pandemic, which also substantially compromised the region’s economy. As a result, there were economic pressures, especially from traders, that led governors and mayors to reduce restrictive measures, even when the pandemic was stable.^33^

Regarding spatial analysis, we used the empirical Bayesian method to smooth the mortality rates to represent the epidemiological scenario more accurately. This method allows the reduction of data fluctuations in small areas. When rates were smoothed, we observed a homogenization of spatial distribution. Considering that the Bayesian rates attribute more influence to neighboring municipalities, the results are more coherent at regional level.^34^

Applying the space–time scanning method, we identified two clusters of high risk of deaths from COVID-19 in the Northeast region. The primary cluster was formed by 776 municipalities in the states of Ceará, Rio Grande do Norte, Paraíba, Pernambuco, and Alagoas, whereas the secondary cluster covered the municipalities in the central-south region of the state of Sergipe and almost all municipalities in the state of Bahia. Notably, the capitals and metropolitan areas of these states are located on the coastal strip of the region. These are cities with the highest population density, the highest population and tourist flow and, consequently, at significantly increased risk of spreading the virus.^12^

Correspondingly, this scenario can explain the dispersion process of COVID-19 from the large urban centers and more developed cities to smaller and inland municipalities.^35^ Corroborating this, a study conducted in Bahia showed that, on the basis of the spatial dispersion pattern of COVID-19, airports and highways in urban centers were responsible for the interiorization of the disease in the state.^36^

In addition, it has been shown that tourism, economic networks, and social mobility are important factors in better understanding the progression of SARS-CoV-2 in different areas.^37^ Most states identified with high mortality and CFR are important tourist destinations with high urban mobility due to economic activity.

Recent studies have shown that high SARS-CoV-2 transmission can be observed in areas with greater socioeconomic vulnerability.^14^^,^^38^^,^^39^ Importantly, most inland municipalities have a high SVI and a low HDI. Even in large urban centers, there are areas of greater poverty, precarious household and sanitary infrastructure, and household clusters such as slums. Unfortunately, these are areas with greater social vulnerability and, consequently, at high risk of transmitting the virus.

Furthermore, these populations usually have a high prevalence of other clinical conditions, such as diabetes, hypertension, and cardiovascular disease, which, along with COVID-19, are related to the worst clinical prognosis of the disease and the highest mortality.^40^ More importantly, less-developed cities do not have adequate hospital infrastructure. Commonly, they have a reduced number of clinics and intensive care units that are exclusively for COVID-19. As a result, the increased flow of patients to hospitals in metropolitan cities can therefore overwhelm the health services.^41^^,^^42^

Likewise, Andrade and colleagues identified high-risk clusters in the central-south region of the state of Sergipe, which has a high population density and low socioeconomic level. Additionally, the state of Sergipe has one of the highest mortality rates in the study period.^6^ We also highlight 33 municipalities in Sergipe that presented a high risk of mortality, which corroborates the state being severely affected by COVID-19. Taken together, these findings demonstrate the relationship of epidemiological indicators with the highest mortality due to COVID-19.^43^

Despite the strengths, our study has some limitations that deserve to be mentioned. The use of secondary public-domain data may underreport deaths, and suspected or undefined cases may not have been included. Additionally, the use of the SVI in Brazil as a composite indicator (by including three dimensions) can mask inequalities between populations. Therefore, further studies should implement techniques to evaluate clusters of cases while simultaneously adjusting for age and sex and other relevant covariates.

Altogether, our analyses demonstrated municipalities with a high mortality and CFR due to COVID-19 in Northeast Brazil. Furthermore, increasing trends in deaths were observed particularly in municipalities with greater SVI. In addition, spatiotemporal analyses identified two high-risk clusters of deaths from COVID-19, mainly in municipalities on the coastal strip of the region, which are areas with the highest population density and tourism flows. Considering all this, we suggest that preventive strategies, such as implementing more restrictive measures to reduce social mobility, social and economic support by the federal government, and widespread vaccination of the population, should be implemented urgently to reduce the number of cases and deaths and avoid the collapse of the health system in Brazil.
